# Genotype frequencies of polymorphic MDR1 variants in the Kazakhstani population

**DOI:** 10.5195/cajgh.2013.118

**Published:** 2014-03-27

**Authors:** Samat Kozhakhmetov, Adil Supiyev, Almagul Kushugulova, Indira Tynybayeva, Alibek Kossumov, Leila Utepova, Saule Saduakhasova, Gulnara Shakhabayeva, Talgat Nurgozhin, Zhaxybay Zhumadilov

**Affiliations:** Center for Life Sciences, Nazarbayev University, Astana, Kazakhstan

**Keywords:** genotype, MDR1, Kazakh population

## Abstract

**Introduction:**

Statins appear to be handled by an ATP-dependent membrane transporter and three SNPs (C1236T (rs1128503), G2677T (rs2032582), and C3435T (rs1045642), which capture the common genetic variation at this locus. Individuals, who carry the T allele at each SNP (i.e., the T-T-T haplotype), have higher systemic exposure to simvastatin.

A triallelic thymine (T) - guanine (G) - adenine (A), which is a point mutation at nucleotide 2677 in exon 22, leads to ABCB1 in a non-synonymous codons (GCT alanine, TCT serine, threonine ACT) at position 893 in a cytoplasmic loop of ATP-dependent membrane transporters.

**Methods:**

Blood samples from healthy individuals were collected in the Republican Diagnostic Center, Astana, Kazakhstan. The research samples included 461 healthy people. Genomic DNA was extracted from peripheral blood using the ‘salting out’ procedure. For the MDR1 exon 21, 2677G>T/A (Ala893Ser/Thr) polymorphism was genotyped by PCR sequencing by the use of dye-terminator (ABI 3730xl sequencer).

**Results:**

The GG allele appeared in 23% of samples, the GA in 6.7%, the GT in 44%, the non-G heterozygote in 4.5%, and the non-G homozygote in 18%. These results are consistent with previously published data. Importantly, the frequency of 2677T alleles in our group was 15.4%. This represents the lowest frequency of this allele compared to published data in different populations. The frequency of the 2677T allele in Asians and Caucasians varies from 38 to 62%, and is 15% for African Americans. On the other hand, the 2677A allele frequency in the Japanese varies from 15 to 22%, and in Caucasians from 2% and 4%. The 2677A allele frequency has been found in 4.6% of samples.

**Conclusions:**

Our study further emphasizes differences between various Asian populations and the importance of repeating this genetic study in different ethnic groups.

